# Dickkopf-1 (DKK1) blockade mitigates osteogenesis imperfecta (OI) related bone disease

**DOI:** 10.1186/s10020-024-00838-3

**Published:** 2024-05-21

**Authors:** Jih-Yang Ko, Feng-Sheng Wang, Wei-Shiung Lian, Fu-Shine Yang, Jeng-Wei Chen, Po-Hua Huang, Chin-Yi Liao, Shu-Jui Kuo

**Affiliations:** 1 Department of Orthopedic Surgery, Kaohsiung Chang Gung Memorial Hospital and Chang Gung University College of Medicine, Kaohsiung City, 833401 Taiwan; 2 Department of Medical Research, Kaohsiung Chang Gung Memorial Hospital and Chang Gung University College of Medicine, Kaohsiung City, 833401 Taiwan; 3 Center for Shockwave Medicine and Tissue Engineering, Kaohsiung Chang Gung Memorial Hospital and Chang Gung University College of Medicine, Kaohsiung City, 833401 Taiwan; 4 School of Medicine, China Medical University, Taichung City, 404328 Taiwan; 5 Department of Orthopedic Surgery, China Medical University Hospital, Taichung City, 404327 Taiwan

**Keywords:** Osteogenesis imperfecta (OI), Dickkopf-1 (DKK1), β-Catenin, T-cell factor 4 (TCF4), Wnt3a, Osteoprotegerin (OPG), Nuclear factor-kappa B ligand (RANKL)

## Abstract

**Background:**

The current treatment of osteogenesis imperfecta (OI) is imperfect. Our study thus delves into the potential of using Dickkopf-1 antisense (DKK1-AS) to treat OI.

**Methods:**

We analysed serum DKK1 levels and their correlation with lumbar spine and hip T-scores in OI patients. Comparative analyses were conducted involving bone marrow stromal cells (BMSCs) and bone tissues from wild-type mice, untreated OI mice, and OI mice treated with DKK1-ASor DKK1-sense (DKK1-S).

**Results:**

Significant inverse correlations were noted between serum DKK1 levels and lumbar spine (correlation coefficient = − 0.679, p = 0.043) as well as hip T-scores (correlation coefficient = − 0.689, p = 0.042) in OI patients. DKK1-AS improved bone mineral density (p = 0.002), trabecular bone volume/total volume fraction (p < 0.001), trabecular separation (p = 0.010), trabecular thickness (p = 0.001), trabecular number (p < 0.001), and cortical thickness (p < 0.001) in OI mice. DKK1-AS enhanced the transcription of collagen 1α1, osteocalcin, runx2, and osterix in BMSC from OI mice (all p < 0.001), resulting in a higher von Kossa-stained matrix area (p < 0.001) in ex vivo osteogenesis assays. DKK1-AS also reduced osteoclast numbers (p < 0.001), increased β-catenin and T-cell factor 4 immunostaining reactivity (both p < 0.001), enhanced mineral apposition rate and bone formation rate per bone surface (both p < 0.001), and decreased osteoclast area (p < 0.001) in OI mice. DKK1-AS upregulated osteoprotegerin and downregulated nuclear factor-kappa B ligand transcription (both p < 0.001). Bone tissues from OI mice treated with DKK1-AS exhibited significantly higher breaking force compared to untreated OI mice (p < 0.001).

**Conclusions:**

Our study elucidates that DKK1-AS has the capability to enhance bone mechanical properties, restore the transcription of osteogenic genes, promote osteogenesis, and inhibit osteoclastogenesis in OI mice.

## Background

Osteogenesis imperfecta (OI) is a rare hereditary bone disorder characterized by severe osteoporosis, deformity, fragility, and recurrent fractures (Ko et al. 2023a; Syu et al. 2022; Faienza et al. 2023; Tyurin et al. 2022). Mutations in the genes encoding type I collagen, abnormalities in collagen folding, or post-transcriptional modifications that disrupt the ultrastructure of collagen in the skeletal microenvironment are implicated in the pathogenesis of OI (Barnes et al. 2010, 2006). Within the array of osteoporosis treatments, bisphosphonates have validated their efficacy in treating OI only under very strict indications and assessment criteria (Dwan et al. 2016). Conversely, the effectiveness of teriparatide, denosumab, and anti-sclerostin agents remains a subject of contention in the realm of OI management. However, none of the treatments have demonstrated universal efficacy, thus underscoring the unmet clinical need for managing OI (Otsuru et al. 2012; Bargman et al. 2010; Bishop et al. 2013).

The Dickkopf (DKK) family consists of four primary secreted glycoproteins, namely DKK1, DKK2, DKK3, and DKK4, each comprising 255–350 amino acids. Notably, DKK1 plays a crucial role as a suppressor of canonical Wnt signaling (Jiang et al. 2022). Modulating the detrimental effects of DKK1 has emerged as an effective approach for preventing bone loss associated with estrogen deficiency, excessive glucocorticoid exposure, and osteoarthritis-related joint damage (Wang et al. 2007, 2008; Weng et al. 2012). Recent evidence has linked recessive OI with missense mutations in the Wnt1 gene (Laine et al. 2013). Furthermore, sclerostin antibody neutralization has shown improvements in bone mass in OI mouse models, indicating the involvement of Wnt signaling components in the pathogenesis of OI (Sinder et al. 2013). Given these findings, it is imperative to investigate the biological roles of DKK1 in OI-related bone loss.

The present study aimed to explore the role of DKK1 in the bone metabolism in OI patients. We would investigate whether modulation of Wnt or DKK1 signaling influenced osteogenic responses in bone marrow stromal cells (BMSCs) derived from OI patients. Furthermore, we examined the effects of DKK1 knockdown on bone mass, microarchitecture, and bone remodeling processes in OI mice.

## Methods

### Enzyme-linked immunosorbent assay (ELISA) and bone mineral density analysis for human participants

This study received approval from the Institutional Review Board of Kaohsiung Chang Gung Memorial Hospital (96-1407B). All participants provided written informed consent. Nine individuals, consisting of 7 females and 2 males, spanning an age range of 18 to 40 years and clinically diagnosed with type I OI, were conscientiously recruited. There is no familial linkage among these 9 individuals. Genetic testing information is accessible for three participants, all of whom exhibited mutations in the Col1A1 gene. The exclusion criteria include recent use of anti-osteoporosis medication, occurrence of fractures within the preceding 3 months, or diagnosis of osteonecrosis within the same timeframe prior to blood sampling. Each OI patient was matched with a healthy adult volunteer specifically recruited for this study of similar age (within a 5-year range) and corresponding gender. A total of 10 to 15 mL of venous blood were procured from each participant and subsequently preserved at a temperature of − 80 °C until analysis. The quantification of serum DKK1 concentrations was achieved employing an ELISA kit (Thermo Fisher Scientific, Waltham, MA, USA), following the manufacturer’s stipulated guidelines. Bone mineral density (BMD) and T-score values within the lumbar spine and hip regions were assessed through dual-energy X-ray absorptiometry (DEXA) (Hologic ODR 4500A, Waltham, MA, USA), following our previous protocol (Kuo et al. 2023). The Spearman correlation coefficient was employed to gauge the strength of correlation between serum DKK1 concentration and T-score values among the OI patients.

### Reverse transcription-quantitative polymerase chain reaction (RT-qPCR) assay

The assessment of gene transcription levels was conducted employing the reverse transcription-quantitative polymerase chain reaction (RT-qPCR) assay following our previous works (Ko et al. 2022a, b, 2023a, b). Tissues were pulverized under liquid nitrogen in the absence of RNase, and total RNA was extracted using the RiboPure RNA purification kit (Thermo Fisher Scientific, Waltham, MA, USA). Subsequently, one microgram of RNA was reverse transcribed into complementary deoxyribonucleic acid (DNA) using the Step One Real-Time PCR System (Thermo Fisher Scientific, Waltham, MA, USA). The threshold cycle (Ct), representing the cycle number required for the fluorescence signal to become detectable, was determined. The mRNA expression levels of the analyzed genes were quantified by the equation of ${2}^{-\Delta \Delta Ct}$.

### Establishment of OI mice animal model, treatment, and blood biochemical analysis

The animal study protocol was granted approval through animal use protocols (No. 2010121415 and No. 2015122306) from the Institutional Animal Care and Use Committee of Kaohsiung Chang Gung Memorial Hospital. Experimental models of OI were established using homozygous oim/oim mice (B6C3Fe a/a-Col1a2oim/J), characterized by a mutation in the collagen 1α2 gene, and were procured from the Jackson Laboratory (Bar Harbor, ME, USA). The comparison group consisted of normal control mice of the B6 strain, obtained from BioLasco Inc (Nangang Dist., Taipei City, Taiwan).

Phosphorothioate-modified DKK1 antisense oligonucleotides, specifically targeting nucleotides 4–21 of the DKK1 mRNA coding region (5ʹ-TACAGATCTTGGACCAGA-3ʹ; DKK1-AS), were synthesized. A sense control oligonucleotide with the same phosphorothioate modification was employed for comparative purposes (5ʹ-TCTGGTCCAAGATCTGAT-3ʹ; DKK1-S). Male mice, aged three months, were administered a daily dose of 50 μg/kg of either DKK1-AS or DKK1-S intraperitoneally for four weeks (Huang et al. 2015). While previous studies have suggested dosages of 20 μg/kg or 50 μg/kg for DKK1-AS (Wang et al. 2007, 2008; Weng et al. 2010, 2012; Huang et al. 2015; Lin et al. 2010), our investigation revealed significant effects only in OI mice treated with 50 μg/kg DKK1-AS as compared to untreated OI mice. Hence, we opted for the dosage of 50 μg/kg in our study.

Each animal received an intraperitoneal injection of 25 μg/mL calcein, enabling the labeling of mineral acquisition status at 3 and 9 days prior to euthanasia. At the culmination of the experiment, all animals were humanely euthanised in preparation for subsequent analysis. Immediately prior to euthanasia, cardiac puncture was conducted, and serum samples were stored at − 80 °C until biochemical analysis was carried out. The levels of serum alanine aminotransferase (ALT), blood urea nitrogen (BUN), and creatinine (CRE) in the mice were assessed using a SPOTCHEM EZ automated biochemical analyzer (SP-4430, Arkray, Kyoto, Japan), following protocols outlined in our prior research (Huang et al. 2022). Body weight was assessed promptly following blood sampling.

### Histomorphometry and immunohistochemistry assays

The intact tibiae were embedded in methylacrylate and prepared as sections for subsequent histomorphometric analyses. Von Kossa staining and tartrate-resistant acid phosphatase (TRAP) cytochemical staining were performed to evaluate various aspects of bone structure and composition. To assess mineral deposition, fluorescence microscopy was used to visualize calcein labeling. Parameters such as mineral apposition rate (MAR) and bone formation rate per bone surface (BFR/BS) were calculated based on the calcein-labeled section (Lian et al. 2021). Mineral apposition rate (MAR) is delineated as the distance between the midpoints or corresponding edges of two consecutive calcein labels, divided by the time elapsed between the midpoints of the labeling periods. Bone formation rate (BFR) mirrors the quantity of mineralised bone generated per unit of time. This parameter is computed as the product of the MAR and the bone surface (Xing et al. 2023; Parfitt et al. 1987). Decalcified tibiae, embedded in paraffin wax, were sectioned and subjected to immunohistochemical staining using specific monoclonal antibodies against β-catenin (Sigma Aldrich, St. Louis, MO, USA) and transcription factor-4 (TCF4) (Sigma Aldrich, St. Louis, MO, USA) to investigate their expression patterns in bone tissue.

### Micro-computed tomography (micro-CT) assay

Micro-computed tomography (micro-CT) of bone tissues and reconstruction of 400 radiographs (9 µm pixel) into transverse and sagittal views of images were carried out employing 1176 Skyscan scanner (Bruker, Billerica, MA, USA) and SKYSCAN^®^ CT-Analysis software. The BMD of the distal femur was assessed upon calibration employing hydroxyapatite phosphate (HA) phantom (750 mgHA/cc, Computerized Imaging Reference Systems, Inc., Norfork, VA, USA). Morphometric parameters of distal femur, including BMD (mg/cm^3^), trabecular bone volume to total volume fraction (BV/TV) (%), trabecular separation (Tb.Sp) (mm), trabecular thickness (Tb.Th) (mm), and trabecular number (Tb.N) (1/mm) were calculated. To analyze cortical thickness at the isthmus of the femur, scanning commenced at around 44% of the total femur length from the distal end and extended about 1 mm proximally (Marulanda et al. 2023).

### Biomechanical analysis

A 3-point bending protocol with a 6-mm jag span was employed to assess the breaking forces of femoral diaphysis using the SHIMADZU EZ-SX Material Test System (Shimadzu Corporation, Kyoto, Japan), as described in our previous study (Wu et al. 2021). The displacement speed of the stainless-steel load (50 N) was set at 2 cm/min.

### Harvest of BMSCs from OI patients

Primary BMSCs were obtained intraoperatively from three OI patients, as previously recruited among the nine patients, ensuring a timeframe not within three months before blood sampling. These cells were subsequently isolated and cultured, advancing to the third passage under osteogenic conditions (0.01 mM dexamethasone, 200 μM ascorbic-acid-2-phosphate, and 10 mM β–glycerophosphate) for around 18 days (Kaneto et al. 2014). Subsequently, cultured BMSCs (1 × 10^6^ cells per well, 6 wells) were administered DKK1 recombinant protein (1000 ng/mL) (Sigma-Aldrich, St. Louis, MO, USA) or Wnt3a recombinant protein (1000 ng/mL) (Sigma-Aldrich, St. Louis, MO, USA) within the osteogenic medium, for 24 h. The levels of expression pertaining to osteogenic genes were quantified via the employment of RT-qPCR.

### Assessment of ex vivo osteogenic potential of BMSCs

BMSCs were harvested from the mouse femur as previously described (Wu et al. 2021; Ma et al. 2024; Xie et al. 2023; Li et al. 2023). Harvested primary BMSCs were mixed with red blood cell lysis buffer (Sigma-Aldrich, St. Louis, MO, USA) to isolate mononuclear cells. Following overnight incubation of mononuclear cells in Dulbecco’s modified Eagle medium supplemented with 10% fetal bovine serum (FBS) (Thermo Fisher Scientific, Waltham, MA, USA), adherent cells were harvested. These cells were subsequently cultured in osteogenic medium (10^5^ cells/well in 24-well plates) (StemPro™ Osteogenesis Differentiation Kit; Thermo Fisher Scientific, Waltham, MA, USA) for passaging every 5–7 days, maintaining a plating density of 100 cells/cm^2^ for around 18 days. The extent of mineralization was assessed employing von Kossa Stain Kits (Abcam, Cambridge, UK), following instructor’s protocol. Calcium mass deposits were stained black by the kit, and the area of von Kossa-stained mineralized matrices (mm^2^/field) in each × 125 magnification field was measured using light microscopy and image analysis software (Carl Zeiss). Six fields were randomly selected from each well, with a total of six wells for each treatment, to quantify the von Kossa-stained area (Ko et al. 2023a).

### Assessment of ex vivo osteoclastogenic potential of osteoclast precursor cells

Primary osteoclast precursor cells were isolated from mouse femurs to explore the impact of DKK1-AS or DKK1-S treatment on the osteoclastogenic potential of the harvested precursors. Nucleated cells were obtained from the bone marrow using RBC lysis buffer (Sigma-Aldrich, St. Louis, MO, USA) and incubated in α-minimum essential medium (MEM) supplemented with 10% FBS and 20 ng/mL macrophage colony-stimulating factor (M-CSF) (R&D Systems, Minneapolis, MN, USA) for 24 h. Macrophages typically exhibit non-adherence to surfaces; thus, non-adherent cells were collected following incubating nucleated cells in M-CSF treatment. Subsequently, 10^5^ collected non-adherent cells per well were seeded into 24-well plates and cultured in osteoclastogenic medium consisting of α-MEM, 10% FBS, 20 ng/mL M-CSF, and 20 ng/mL receptor activator of nuclear factor kappa-B ligand (RANKL) (R&D Systems, Minneapolis, MN, USA) for 7 days. Osteoclasts were identified using TRAP staining, and six × 125 magnification fields were randomly selected from each well, with a total of six wells for each treatment, to quantify osteoclasts using image analysis software (Carl Zeiss) (Wang et al. 2013; Zhang et al. 2024).

### Statistical analyses

The experiments were all conducted thrice in total. All statistical analyses were performed by employing GraphPad Prism v5.0 (GraphPad Software Inc., San Diego, CA, USA), and all values were shown as the mean ± S.D. Statistical comparisons were performed by Student’s t-test or one-way analysis of variance (ANOVA) (two-tail) followed by post-hoc Bonferroni testing. The statistical difference was deemed significant if the p-value was < 0.05 (Chan et al. 2022; Chen et al. 2020).

## Results

**Fig. 1:**
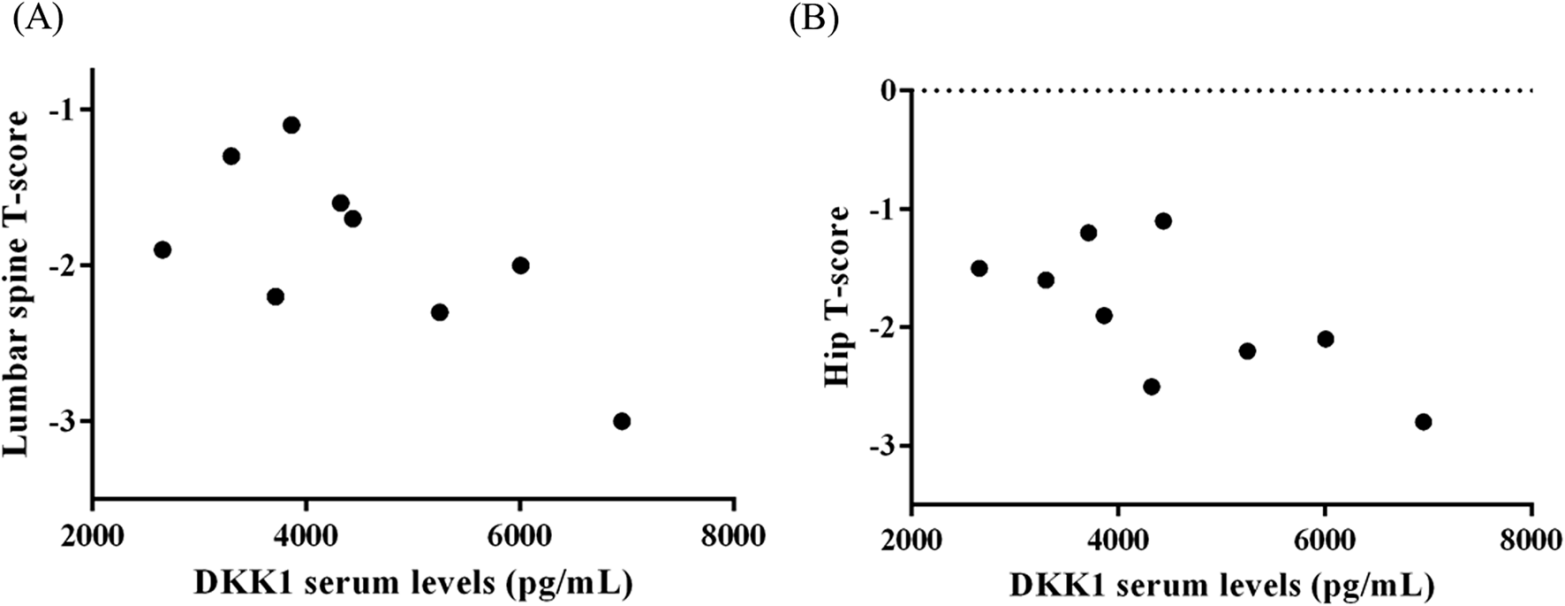
The illustration for the association between serum DKK1 levels and (**A**) lumbar spine T-score and (**B**) hip T-score among OI patients

Nine individuals, comprising 7 females and 2 males, spanning an age range of 18 to 40 years and diagnosed with type I OI, were recruited. Each OI patient was paired with a healthy adult volunteer, aligning their age within a 5-year range and ensuring gender concordance. The median serum DKK1 values were determined to be 4377.83 (3505.26, 5627.39) pg/mL for the OI group and 1992.50 (1797.87, 2403.35) pg/mL for the control group, respectively (p < 0.001). The Spearman correlation coefficient (r) values denoting the association between lumbar spine T-score and serum DKK1 levels, as well as hip T-score and serum DKK1 levels among OI patients, were − 0.679 (p = 0.043) and − 0.689 (p = 0.042), respectively (Fig. 1).

**Fig. 2:**
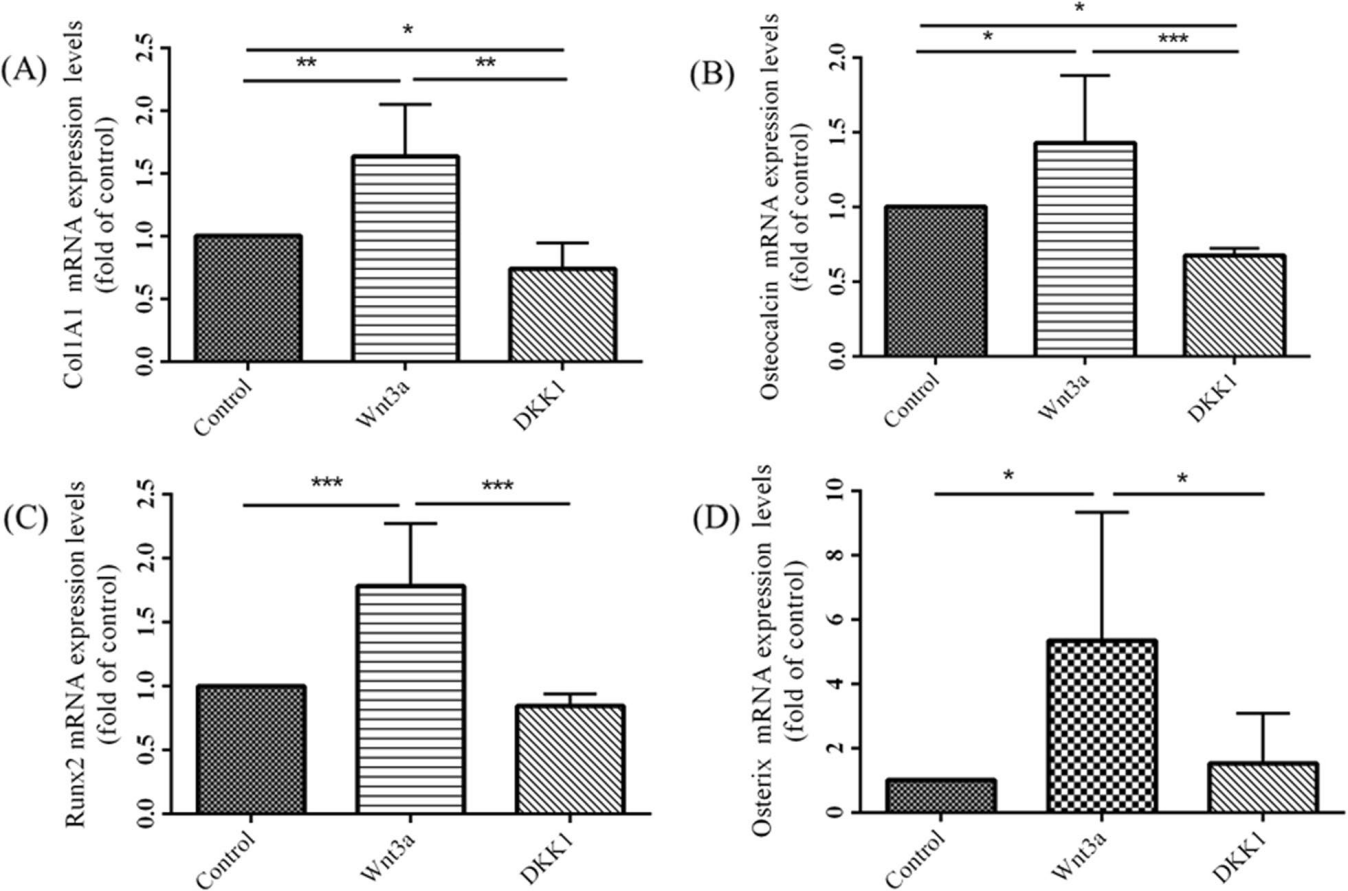
The mRNA expression levels of (**A**) collagen 1α1, (**B**) osteocalcin, (**C**) runx2, and (**D**) osterix in primary BMSCs obtained from OI patients intraoperatively without treatment, treated with Wnt3a recombinant protein, or treated with DKK1 recombinant protein (*p < 0.05, **p < 0.01, and ***p < 0.001 by post-hoc analysis)

Primary BMSCs were procured from OI patients who underwent osteotomy surgeries. These BMSCs were subsequently cultured in an osteogenic medium, concomitant with the administration of either Wnt3a recombinant protein or DKK1 recombinant protein. In contrast to untreated BMSCs and those treated with DKK1 recombinant protein, BMSCs treated with Wnt3a recombinant protein displayed heightened transcription levels of four selected osteogenesis-related genes, comprising collagen 1α1, osteocalcin, runx2, and osterix (Geng et al. 2024; Zhou et al. 2024; Luo et al. 2023). Furthermore, compared to untreated BMSCs, DKK1 recombinant protein-treated BMSCs demonstrated even lower expression of collagen 1α1 and osteocalcin (p < 0.001 for collagen 1α1, osteocalcin, and runx2; p = 0.016 for osterix) (Fig. 2).

**Fig. 3:**
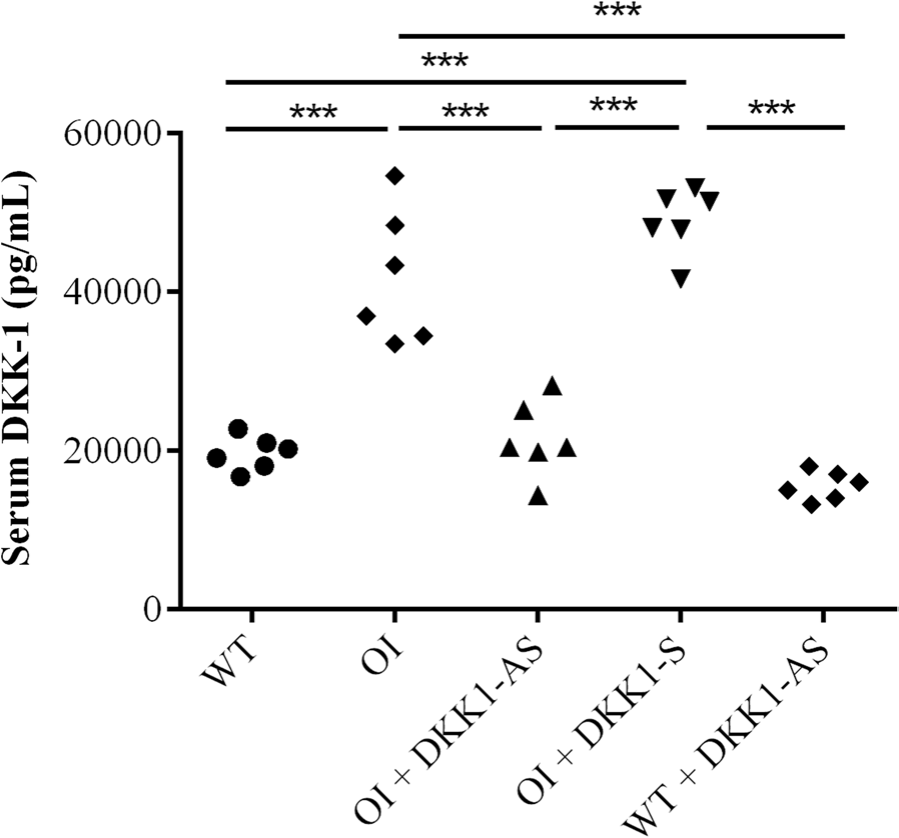
The serum levels of DKK1 measured in wild-type mice, untreated OI mice, OI mice treated with either DKK1-AS or DKK1-S, and wild-type mice treated with DKK1-AS. The sample size for each group was n = 6, with each data point in the figure representing the average of three experiments (***p < 0.001 by post-hoc analysis)

In order to investigate the impact of DKK1 on bone metabolism in OI mice, a protocol entailing the administration of either DKK1-AS or DKK1-S was instituted for four weeks in the ensuing experiments. The serum concentrations of DKK1 showed a significant difference, with higher levels observed in OI mice compared to WT mice and both WT and OI mice treated with DKK1-AS. Additionally, OI mice treated with DKK1-S also exhibited significantly elevated serum DKK1 levels compared to WT mice and both WT and OI mice treated with DKK1-AS (p < 0.001) (Fig. 3).

**Fig. 4:**
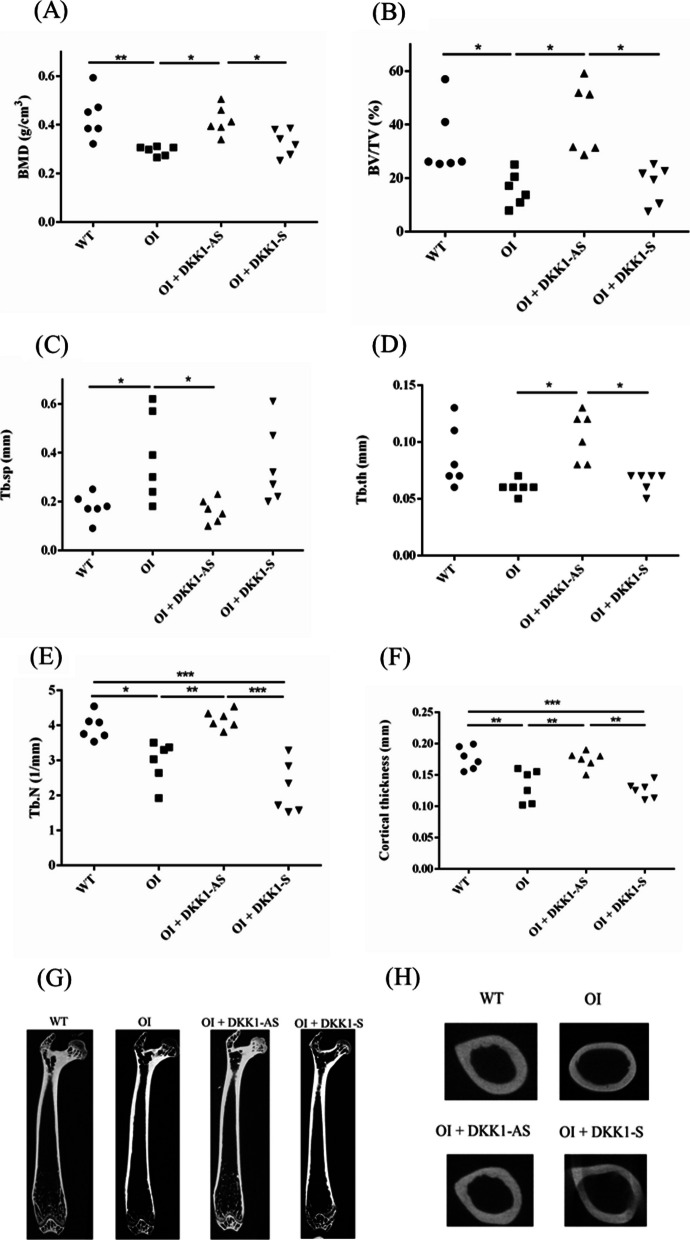
The morphometric parameters of femur samples obtained from wild-type mice, untreated OI mice, and OI mice treated with either DKK1-AS or DKK1-S. **A** BMD: bone mineral density (mg/cm^3^); **B** BV/TV: trabecular bone volume to total volume fraction (%); **C** Tb.sp: trabecular separation (mm); **D** Tb.th: trabecular thickness (mm); **E** Tb.N: trabecular number (1/mm) for distal femur, and **F** cortical thickness (mm) for femoral diaphysis. The sample size for each group was n = 6, with each data point in the figure representing the average of three experiments (*p < 0.05, **p < 0.01, and ***p < 0.001 by post-hoc analysis). The representative micro-CT images of the (**G**) frontal section of whole femur and (**H**) axial section of femoral diaphysis extracted from four groups of mice

The morphometric parameters obtained from micro-CT analysis were compared among wild-type mice, untreated OI mice, OI mice treated with DKK1-AS, and OI mice treated with DKK1-S. In comparison to wild-type mice, untreated OI mice exhibited lower BMD (mg/cm^3^) and lower BV/TV (%). However, OI mice treated with DKK1-AS showed significantly higher BMD and BV/TV values compared to both untreated OI mice and DKK1-S treated OI mice (p = 0.002 for BMD; p < 0.001 for BV/TV). The untreated OI mice displayed higher trabecular separation (Tb.sp) (mm) compared to both wild-type mice and DKK1-AS treated OI mice (p = 0.010). On the other hand, OI mice treated with DKK1-AS exhibited higher trabecular thickness (Tb.th) (mm) compared to both untreated OI mice and DKK1-S treated OI mice (p = 0.001). In comparison to wild-type mice, both untreated and DKK1-S treated OI mice displayed lower trabecular number (Tb.N) (1/mm). However, the OI mice treated with DKK1-AS exhibited higher Tb.N compared to both untreated and DKK1-S treated OI mice (p < 0.001). Regarding cortical thickness (mm) along the femoral diaphysis, wild-type mice demonstrated greater cortical thickness compared to both untreated OI mice and those treated with DKK1-S. Conversely, OI mice treated with DKK1-AS exhibited higher cortical thickness compared to both untreated OI mice and those treated with DKK1-S (p < 0.001) (Fig. 4).

**Fig. 5:**
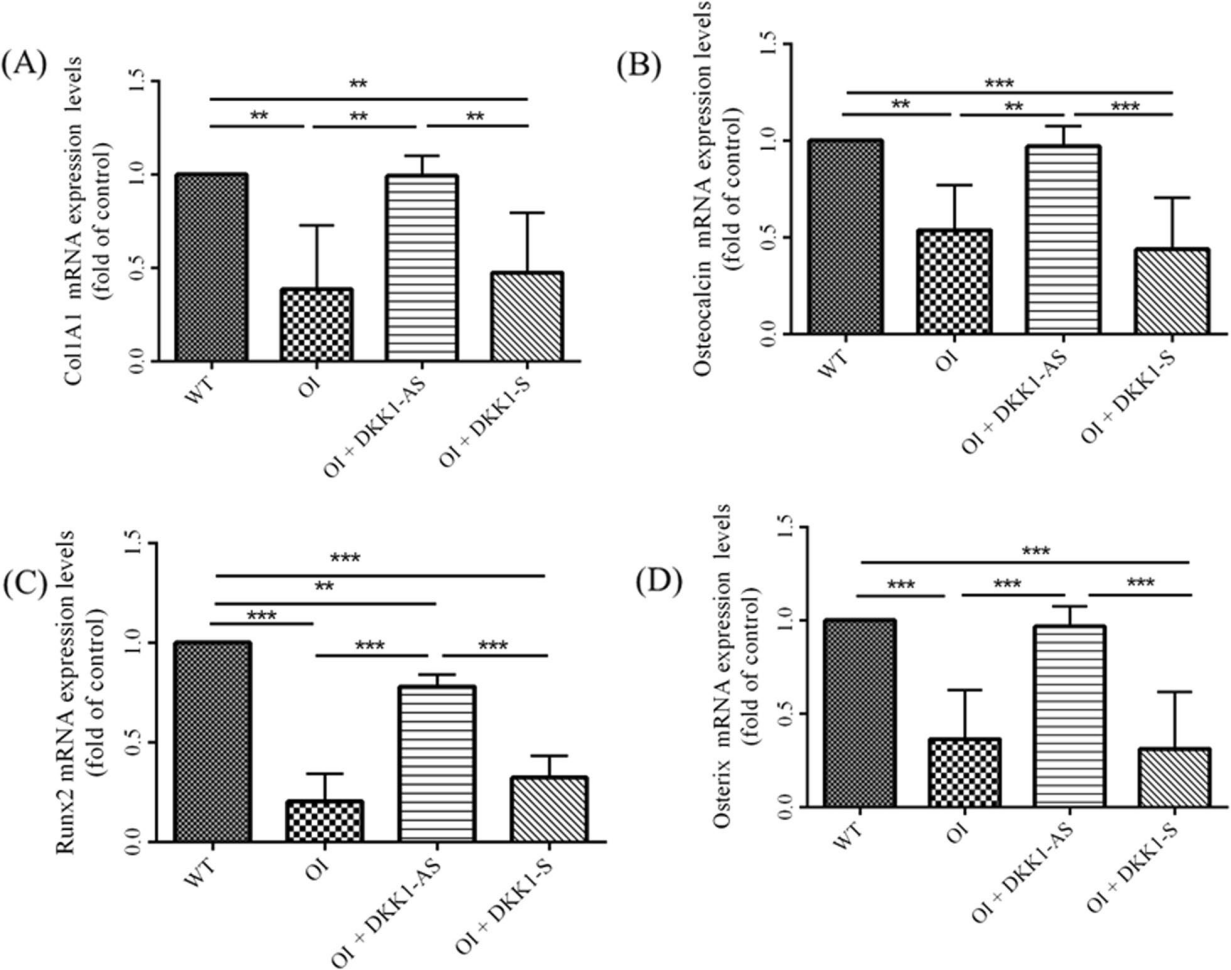
The mRNA expression levels of (**A**) collagen 1α1, (**B**) osteocalcin, (**C**) runx2, and (**D**) osterix in primary BMSCs obtained from wild-type mice, OI mice without treatment, and OI mice treated with either DKK1-AS or DKK1-S (**p < 0.01 and ***p < 0.001 by post-hoc analysis)

**Fig. 6:**
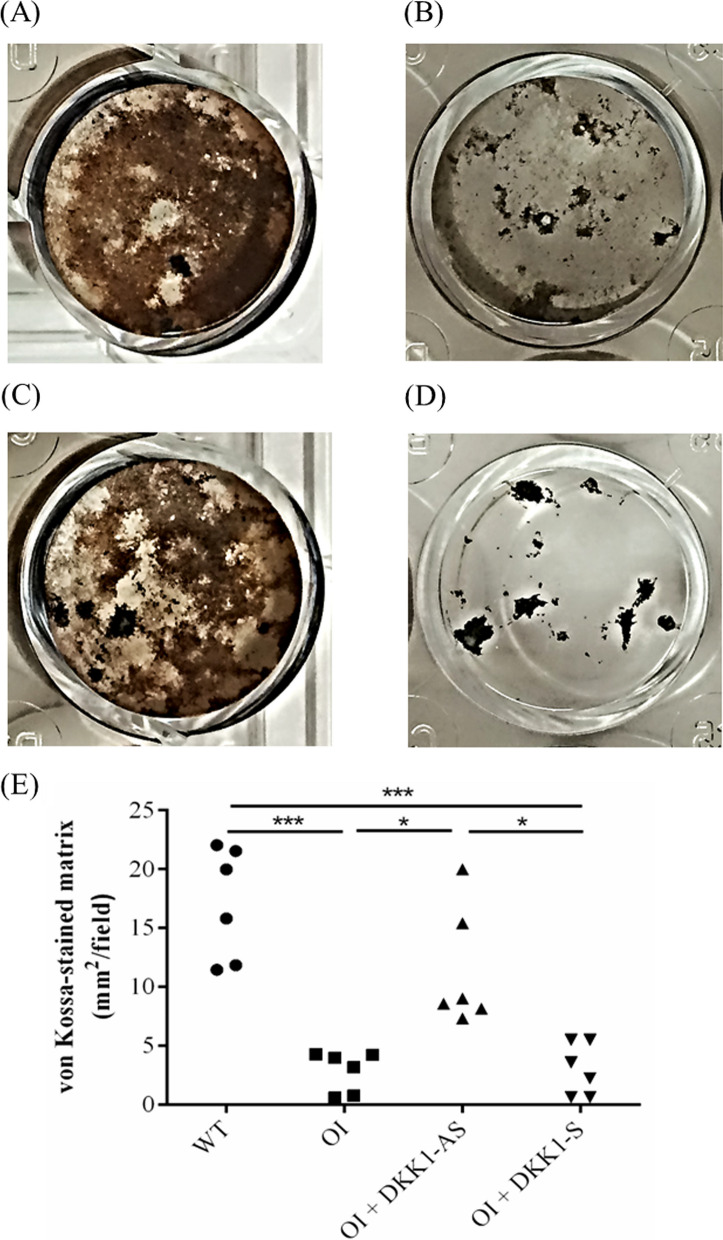
The capacity of ex vivo osteogenic differentiation of BMSCs. Illustration of von Kossa-stained region for the BMSCs harvested from (**A**) wild-type mice; (**B**) untreated OI mice; OI mice treated with either (**C**) DKK1-AS or (**D**) DKK1-S and the (**E**) quantification figure. The surface area for each well was 1.9 cm^2^. The sample size for each group was n = 6, with each data point in the figure representing the average of three experiments (*p < 0.05 and ***p < 0.001 by post-hoc analysis)

Primary BMSCs extracted from the four mouse groups were cultured in an osteogenic medium to assess the influence of DKK1-AS and DKK1-S on the osteogenic differentiation potential of the harvested BMSCs. Expression levels of collagen 1α1, osteocalcin, runx2, and osterix were observed to be diminished in primary BMSCs derived from OI mice compared to those from wild-type mice. However, the application of DKK1-AS significantly ameliorated these suppressive effects, resulting in the restoration of osteogenic gene expression (p < 0.001 for all four genes) (Fig. 5). Furthermore, BMSCs isolated from OI mice exhibited a pronounced reduction in the von Kossa-stained matrix area under osteogenic differentiation condition compared to BMSCs derived from wild-type mice, indicative of compromised osteogenic differentiation. In contrast, BMSCs procured from OI mice treated with DKK1-AS demonstrated a substantial increase in the von Kossa-stained matrix area under osteogenic differentiation conditions when compared to BMSCs obtained from untreated OI mice. This observation suggests a partial restoration of osteogenic activity due to DKK1-AS treatment (p < 0.001) (Fig. 6). These findings highlight the potential of DKK1-AS to restore the osteogenic capacity of BMSCs acquired from OI mice.

**Fig. 7:**
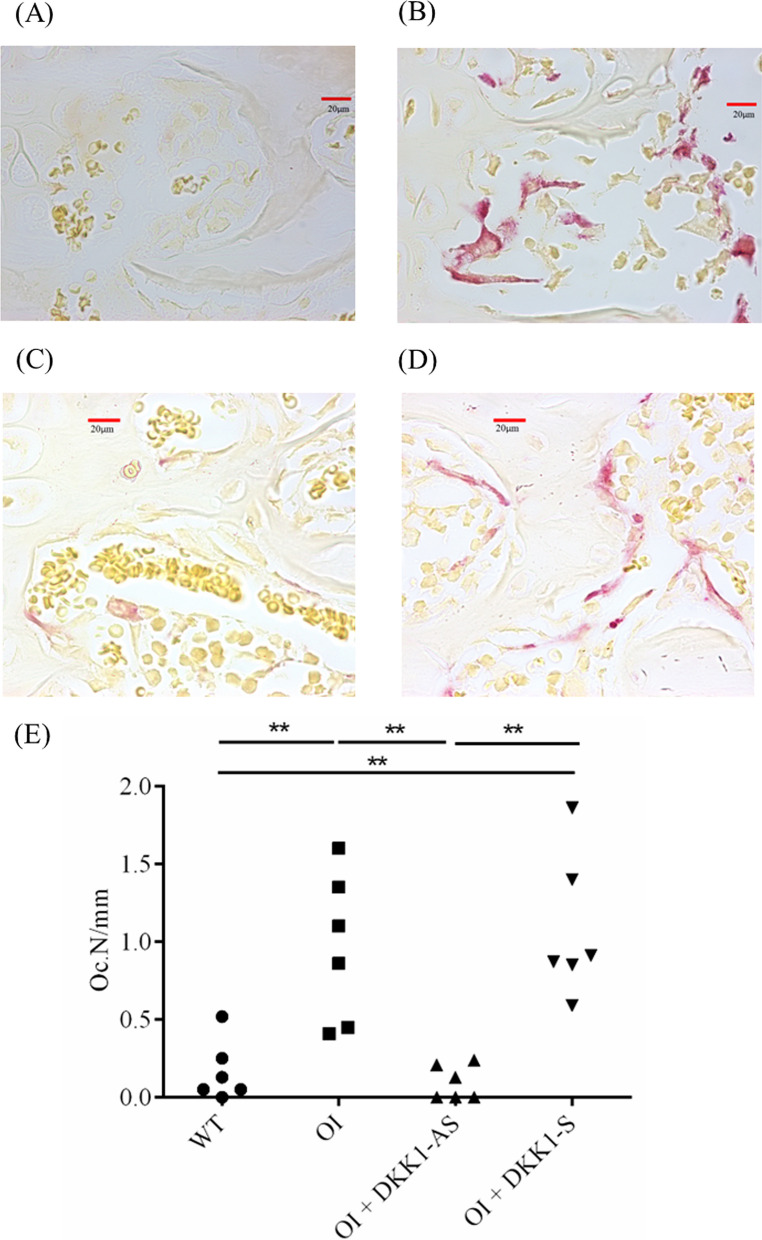
Immunohistochemical images of TRAP in bone tissue harvested from (**A**) wild-type mice; (**B**) untreated OI mice; OI mice treated with either (**C**) DKK1-AS or (**D**) DKK1-S and the (**E**) quantification figure. The sample size for each group was n = 6, with each data point in the figure representing the average of three experiments (**p < 0.01 by post-hoc analysis)

Our study aimed to investigate the effects of DKK1-AS and DKK1-S treatment on bone formation and resorption dynamics within the skeletal microenvironment of OI mice. Histological examination using TRAP staining revealed that bone tissues from both untreated and DKK1-S treated OI mice exhibited a markedly higher osteoclast number (Oc.N) compared to those from wild-type mice. Similarly, bone tissues from DKK1-AS treated OI mice displayed a significantly lower Oc.N when compared to both untreated and DKK1-S treated OI mice (p < 0.001) (Fig. 7).

**Fig. 8:**
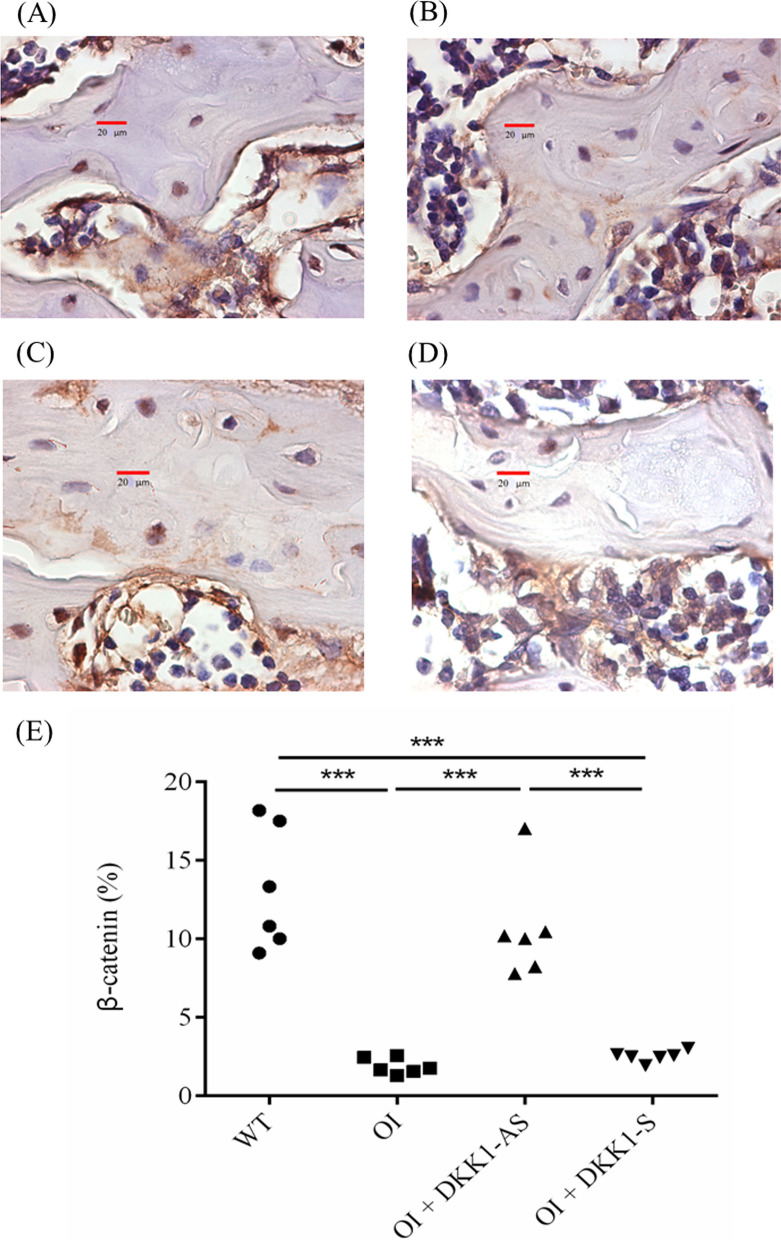
Immunohistochemical images of β-catenin in bone tissue harvested from (**A**) wild-type mice; (**B**) untreated OI mice; OI mice treated with either (**C**) DKK1-AS or (**D**) DKK1-S and the (**E**) quantification figure. The sample size for each group was n = 6, with each data point in the figure representing the average of three experiments (***p < 0.001 by post-hoc analysis)

**Fig. 9:**
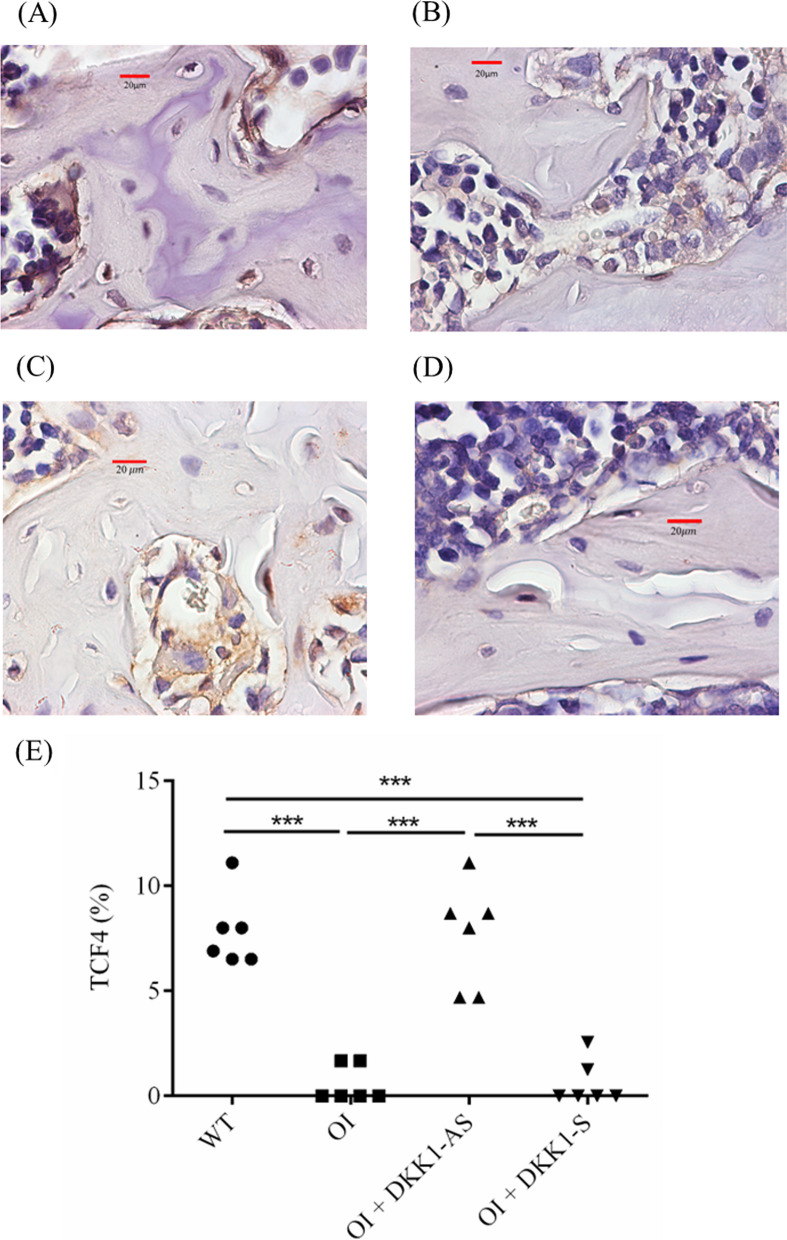
Immunohistochemical images of transcription factor-4 (TCF4) in bone tissue harvested from (**A**) wild-type mice; (**B**) untreated OI mice; OI mice treated with either (**C**) DKK1-AS or (**D**) DKK1-S and the (**E**) quantification figure. The sample size for each group was n = 6, with each data point in the figure representing the average of three experiments (***p < 0.001 by post-hoc analysis)

**Fig. 10:**
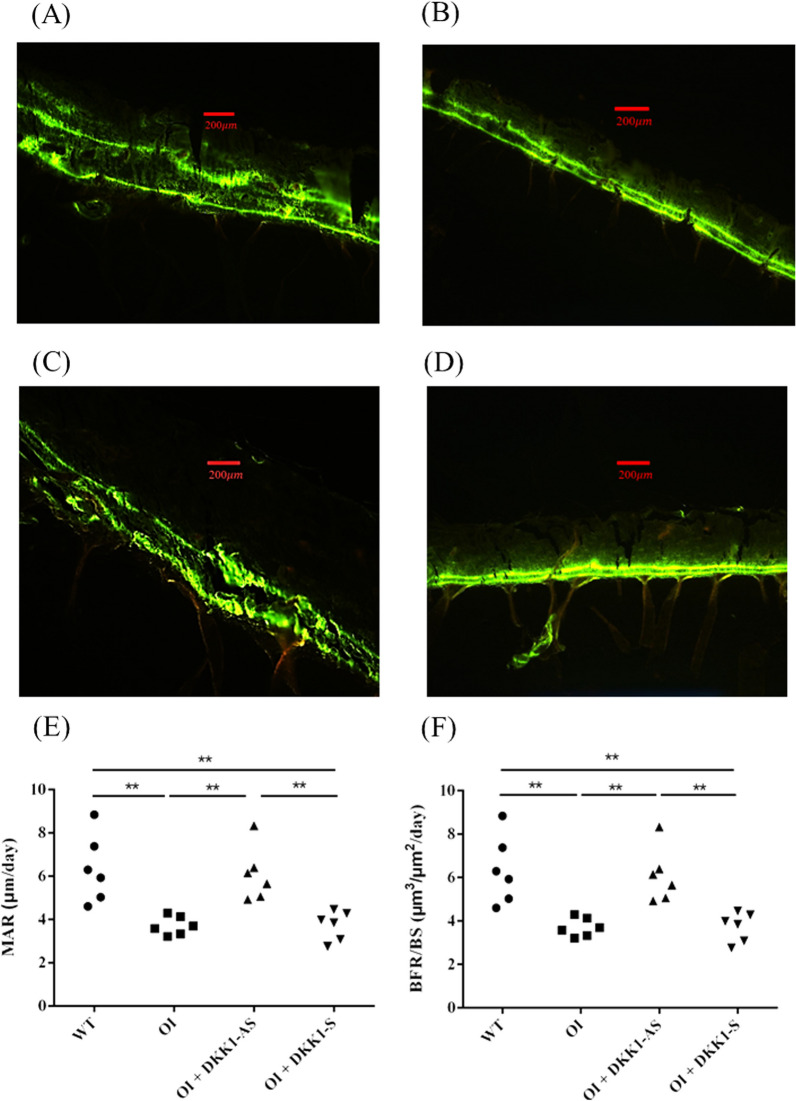
The representative calcein-labeled sections for bone tissues obtained from (**A**) wild-type mice; (**B**) untreated OI mice; OI mice treated with either (**C**) DKK1-AS or (**D**) DKK1-S. The (**E**) mineral apposition rate (MAR) and (**F**) bone formation rate per bone surface (BFR/BS) for the bone tissues obtained from all experiment groups. The sample size for each group was n = 6, with each data point in the figure representing the average of three experiments (**p < 0.01 by post-hoc analysis)

It was observed that bone tissues extracted from both untreated and DKK1-S treated OI mice demonstrated a notably diminished immunostaining reactivity of β-catenin and TCF4, a contrast to the bone tissues derived from their wild-type counterparts. Intriguingly, the bone tissues obtained from DKK1-AS treated OI mice exhibited markedly heightened β-catenin and TCF4 immunoreactivities in comparison to the bone tissue from both untreated and DKK1-S treated OI mice (p < 0.001 for both β-catenin and TCF4) (Figs. 8, 9). The bone tissues sourced from wild-type mice exhibited notably higher MAR and BFR/BS values in comparison to those acquired from both untreated and DKK1-S treated OI mice. Similarly, bone tissues from OI mice treated with DKK1-AS demonstrated significantly higher MAR and BFR/BS measurements compared to bone tissues from both untreated and DKK1-S treated OI mice (p < 0.001 for both MAR and BFR/BS) (Fig. 10).

**Fig. 11:**
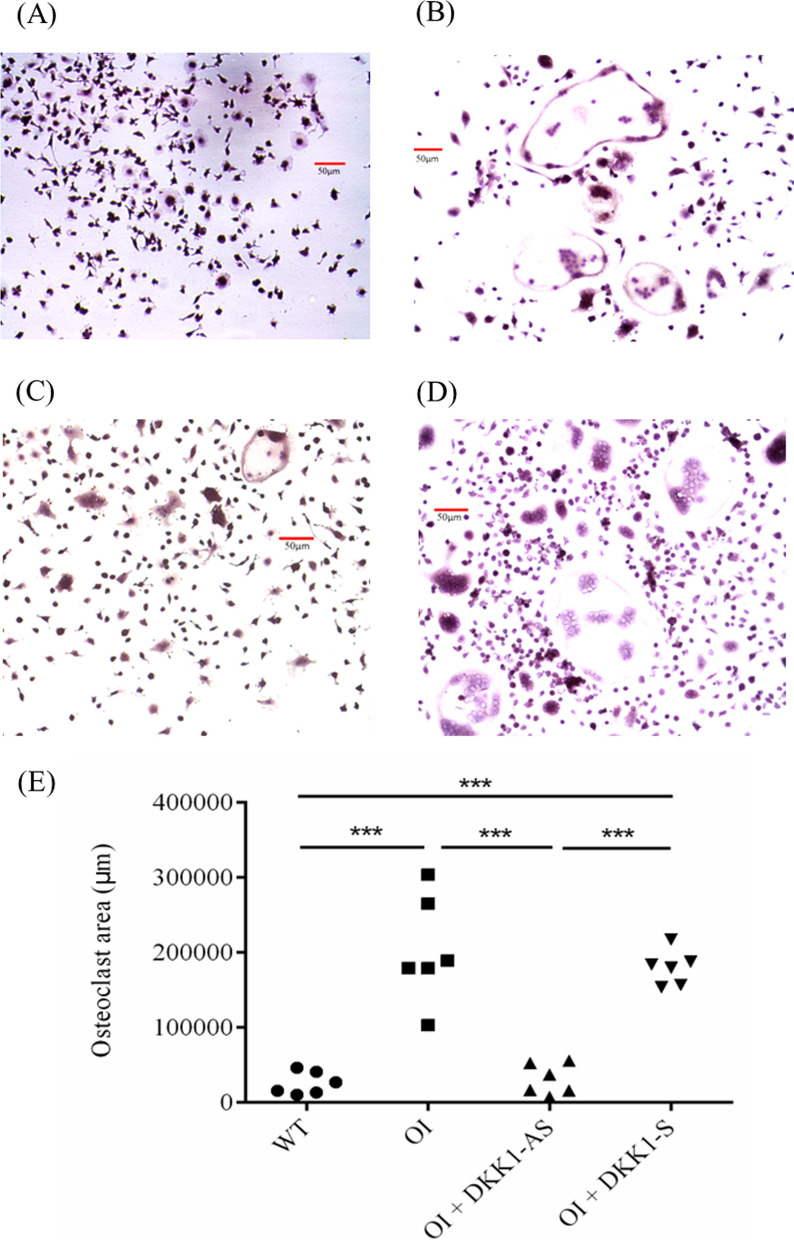
The capacity of ex vivo osteoclastogenic differentiation of bone marrow osteoclast precursor cells. Illustration of TRAP-stained region for the precursor cells harvested from (**A**) wild-type mice; (**B**) untreated OI mice; OI mice treated with either (**C**) DKK1-AS or (**D**) DKK1-S exposed to an osteoclastogenic medium and the (**E**) quantification figure. The sample size for each group was n = 6, with each data point in the figure representing the average of three experiments (***p < 0.01 by post-hoc analysis)

To further elucidate the effects of DKK1-AS or DKK1-S treatment on osteoclast differentiation, we isolated primary bone marrow macrophage precursor cells and subjected them to incubation in an osteoclastogenic medium. The differentiation potential of these precursors into osteoclasts, quantified through the osteoclast area, exhibited a noteworthy increase for precursor cells derived from untreated OI mice in contrast to those from wild-type mice and OI mice subjected to DKK1-AS treatment. Similarly, the osteoclastic differentiation potential of precursor cells from DKK1-S treated OI mice was notably higher compared to those from wild-type mice and OI mice treated with DKK1-AS (p < 0.001) (Fig. 11).

**Fig. 12:**
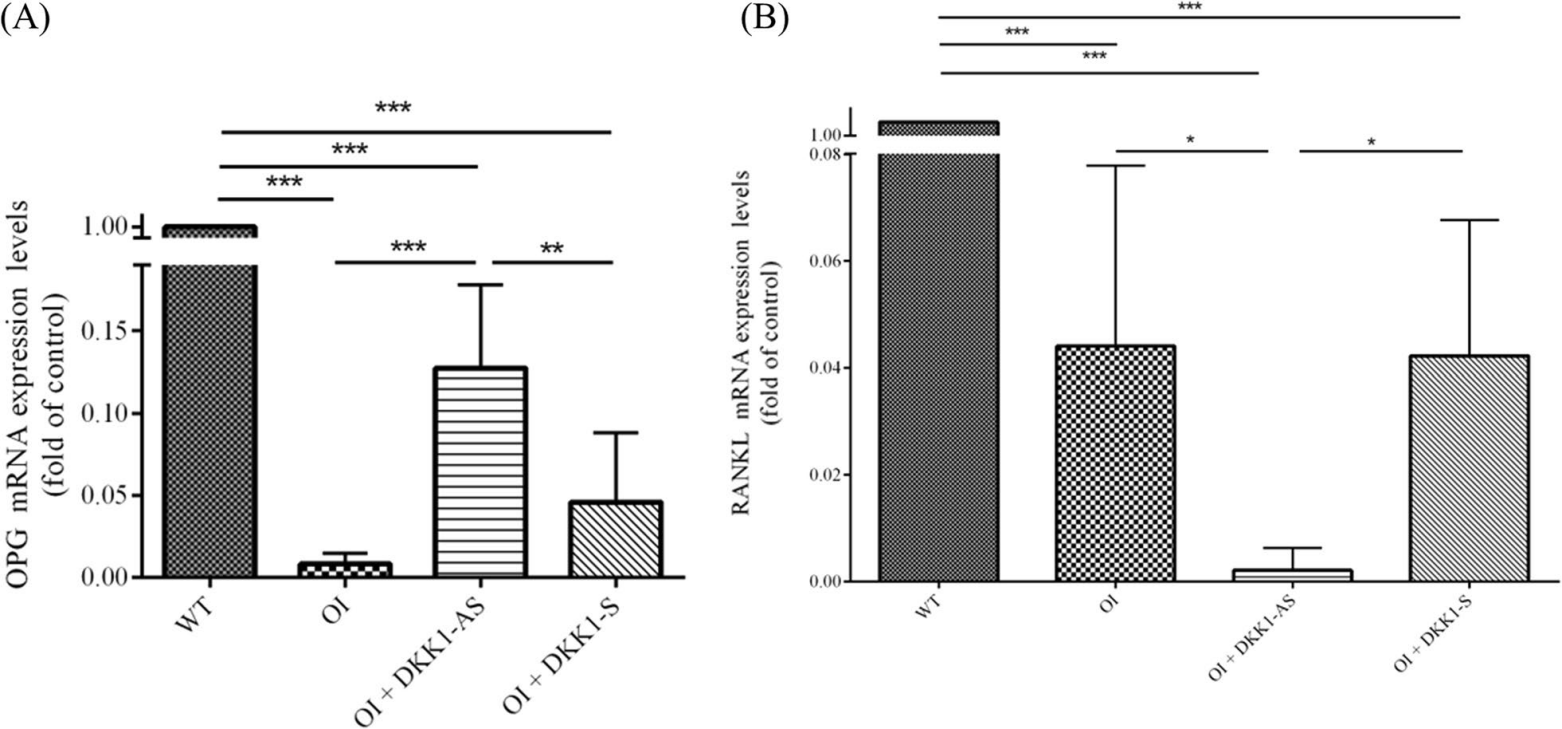
The mRNA expression levels of (**A**) OPG and (**B**) RANKL in bone tissues obtained from wild-type mice, OI mice without treatment, and OI mice treated with either DKK1-AS or DKK1-S (*p < 0.05, **p < 0.01, and ***p < 0.001 by post-hoc analysis)

The expression of osteoprotegerin (OPG) was markedly higher in bone tissues from OI mice treated with DKK1-AS in comparison to bone tissues from both untreated and DKK1-S treated OI mice. Conversely, the expression level of RANKL was significantly lower in bone tissues from OI mice treated with DKK1-AS as opposed to bone tissues from untreated OI mice and from OI mice treated with DKK1-S (p < 0.001 for both OPG and RANKL) (Fig. 12).

**Fig. 13:**
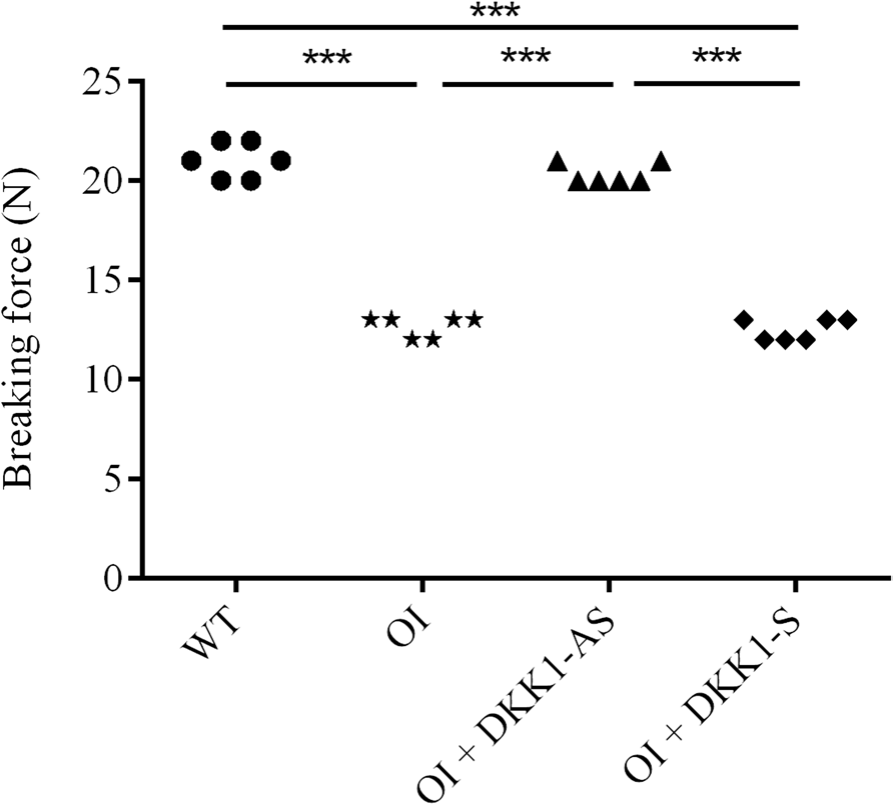
The breaking force of femoral diaphysis in femoral shaft specimens obtained from wild-type mice, untreated OI mice, and OI mice treated with either DKK1-AS or DKK1-S under a 3-point bending protocol. The sample size for each group was n = 6, with each data point in the figure representing the average of three experiments (***p < 0.01 by post-hoc analysis)

A material testing system was employed to assess the maximum breaking forces of bone specimens under three-point bending. Bone tissues from both untreated OI mice and those treated with DKK1-S demonstrated a notably lower breaking force compared to those from wild-type mice. Conversely, bone tissues from OI mice treated with DKK1-AS exhibited a significantly higher breaking force when compared to both untreated OI mice and those treated with DKK1-S (p < 0.001) (Fig. 13).

**Fig. 14:**
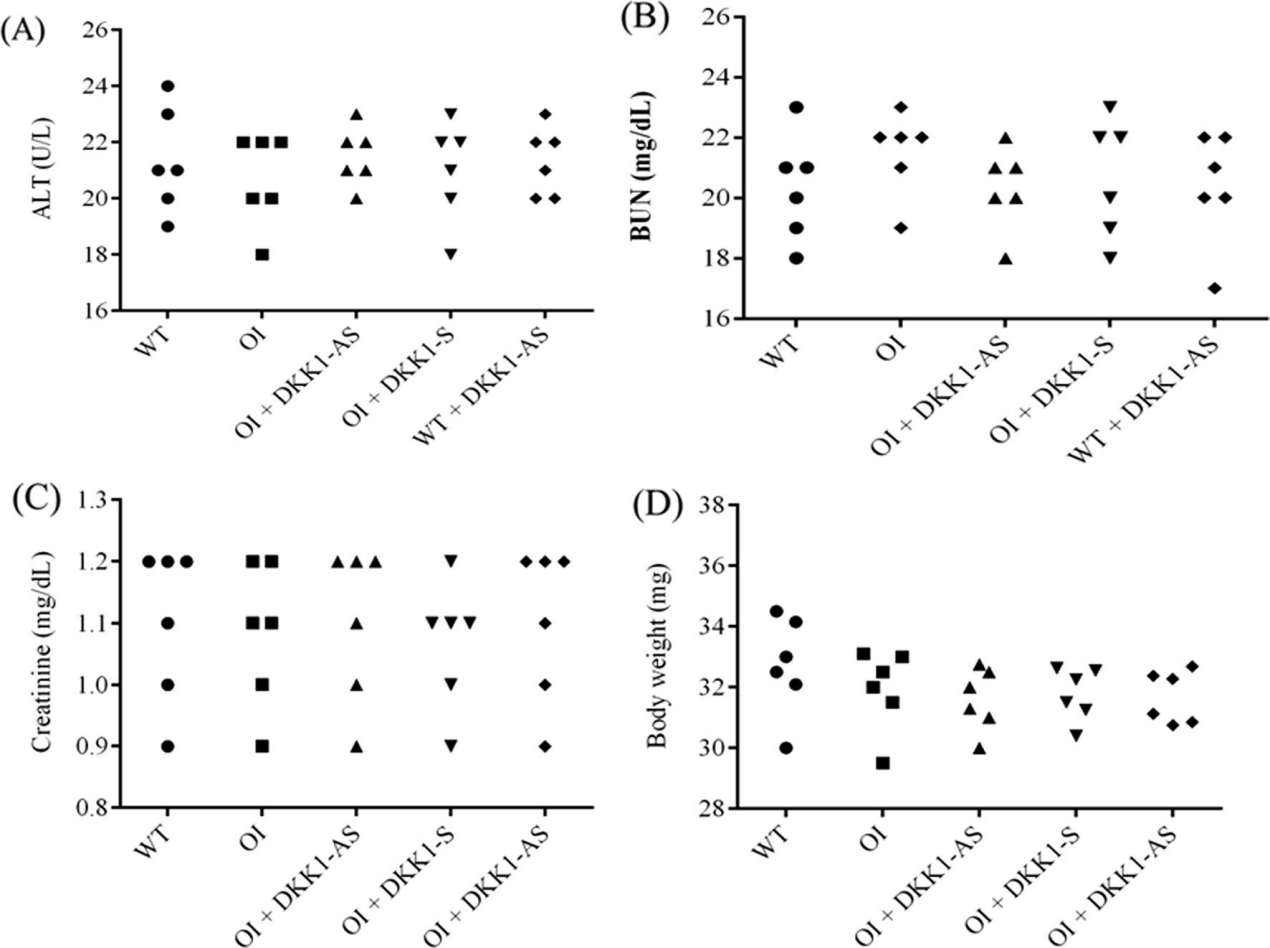
The serum levels of (**A**) ALT, (**B**) BUN, and (**C**) creatinine as well as (**D**) body weight were measured in wild-type mice, untreated OI mice, OI mice treated with either DKK1-AS or DKK1-S, and wild-type mice treated with DKK1-AS. The sample size for each group was n = 6, with each data point in the figure representing the average of three experiments

Ultimately, our aim is to explore whether oligonucleotide treatment might result in hepatic or renal injuries. Our findings indicate that oligonucleotide treatment did not significantly alter serum ALT (p = 0.703), BUN (p = 0.903), and CRE (p = 0.991) levels. The body weight exhibited no variance among the five groups (p = 0.495) (Fig. 14).

## Discussion

In our study, we have demonstrated a clear inverse correlation between serum DKK1 levels and both lumbar spine and hip T-scores among OI patients. Furthermore, the expression of osteogenic genes in BMSCs extracted from OI patients and exposed to Wnt3a recombinant protein exhibited a marked increase in comparison to untreated BMSCs and those treated with DKK1 recombinant protein. The inferior morphometric parameters in OI mice, as observed via micro-CT analyses, exhibited an ameliorative response upon administration of DKK1-AS. The diminished expression levels of osteogenic genes within BMSCs harvested from OI mice, which were notably lower than those of the wild-type counterparts, displayed partial restoration upon treatment with DKK1-AS. The compromised osteogenic capacity of BMSCs and the heightened osteoclastogenic propensity of osteoclast precursor cells sourced from untreated OI mice, in comparison to the respective cells from wild-type mice, were both mitigated through the application of DKK1-AS. DKK1-AS exhibited the ability to quell the elevated osteoclast count evident in bone tissue derived from untreated OI mice, relative to the bone tissue of wild-type mice. Furthermore, the compromised expression of β-catenin and TCF4, relative to wild-type bone tissue, was reinstated by DKK1-AS treatment. The decreased MAR and BFR/BS values evident in bone tissues of untreated OI mice, when contrasted with bone tissues from wild-type mice, experienced a revitalization through DKK1-AS intervention. Notably, the administration of DKK1-AS to OI mice engendered elevated OPG expression and suppressed RANKL expression within bone tissues. These findings are novel and previously unreported. Collectively, our study underscores the therapeutic promise of DKK1-AS in addressing the challenges of OI-related bone ailments.

Currently, a definitive remedy for OI remains elusive. While bisphosphonates improve BMD in OI patients, their effect is primarily recognised in reshaping compressive vertebrae in children with OI only, and bisphosphonates show no effect in adults (Dwan et al. 2016; Li et al. 2019). There is no robust study demonstrating the efficacy of denosumab in reducing fracture incidence and deformities (Hoyer-Kuhn et al. 2016). Teriparatide’s viability is encumbered within the context of OI children, owing to the apprehension stemming from the potential manifestation of osteosarcoma, an observation gleaned from animal studies (Vahle et al. 2002). In the realm of anti-sclerostin agents, Glorieux et al. expounded on the application of BPS804 in adults with moderate OI, demonstrating that BPS804 increased BMD, reduced resorption, and stimulated bone formation (Glorieux et al. 2017). Another study by Uehara et al. presented a case report detailing the successful management of severe osteoporosis in a 64-year-old man with type I OI. Treatment with Romosozumab for one year resulted in increased BMD and elevated bone formation markers (Uehara et al. 2022). A subsequent case report underscored the advantageous outcomes of Romosozumab in a 52-year-old woman diagnosed with type I OI. Despite previous unsuccessful alendronate therapy, she witnessed a 10.3% increase in BMD of the spine and a 5.4% increase in BMD of the right hip. Additionally, the trabecular bone score exhibited a 5.2% increase following Romosozumab treatment (Dattagupta and Petak 2023). The studies examining the administration of anti-sclerostin agents to OI patients are limited, comprising only three involving human subjects, none of which specifically target children, with two being case reports. These unmet clinical requirements have compelled us to seek alternative therapeutic avenues capable of enhancing the bone integrity of individuals afflicted by OI.

Our team has been dedicated to exploring the therapeutic potential of DKK1 inhibition for musculoskeletal disorders. Through our studies, we have observed that DKK1-AS effectively counteracted the suppressive effects of ovariectomy on bone weight, mineral content, density, and peak load of femurs, while also reducing osteoclast differentiation and increasing osteoblast number. This resulted in the mitigation of trabecular bone loss and osteoclast number in ovariectomized rats (Wang et al. 2007). Furthermore, in the context of glucocorticoid-induced bone loss, DKK1-AS treatment alleviated reductions in bone density, trabecular volume, and osteoblast function, while also inhibiting adipocyte volume in the bone marrow and modulating key signaling pathways involved in osteoblast survival and differentiation (Wang et al. 2008). In our investigation of osteonecrosis of the femoral head (ONFH), individuals with ONFH exhibited heightened DKK1 expression in bone tissue and serum, alongside increased apoptosis markers. Additionally, serum DKK1 levels positively correlated with ONFH severity. We also demonstrated the efficacy of plasmids encoding DKK1 RNA interference in mitigating dexamethasone-induced apoptosis in BMSCs derived from ONFH patients (Ko et al. 2010). In the realm of osteoarthritis therapy, our research focused on the role of DKK1 in OA joints, revealing increased vascularity and expression of angiogenic factors and proteinases in synovial tissues from OA patients, alongside elevated DKK1 levels. Through in vivo interference with DKK1 neutralizgn antibody and DKK1-AS treatment, we observed reductions in angiogenic factors and proteinases expression, synovial vascularity, cartilage degradation, and bone density loss in OA knees, suggesting the potential of DKK1 inhibition as a therapeutic approach for reducing OA-induced synovitis and joint deterioration while alleviating cartilage destruction and subchondral bone damage (Weng et al. 2012, 2010). These promising outcomes have motivated us to further investigate DKK1 inhibition in the treatment of OI.

While the direct incorporation of DKK1 inhibition into OI treatment remains hitherto uncharted, antecedent works have delved into the direct and indirect contributions of DKK1 within the ambit of OI pathogenesis. One Italian study encompassed thirteen postmenopausal women diagnosed with type I OI. These individuals, who had undergone neridronate treatment for a minimum of two years and faced new vertebral fractures, were subsequently subjected to an 18-month teriparatide treatment. The study unveiled that no substantial increases occurred in hip BMD over the study duration. The authors also showed serum DKK1 levels demonstrated a progressive and statistically significant increase during the teriparatide treatment. The authors noted that, within type I OI patients receiving teriparatide, the enhancements in BMD were somewhat less conspicuous compared to the improvements observed in postmenopausal or senile osteoporosis patients undergoing teriparatide treatment for a comparable span. This context sparks intriguing speculation regarding the potential correlation between the less discernible therapeutic effects of teriparatide in OI patients and the concurrent elevation in DKK1 levels (Gatti et al. 2013). In G. Brunetti et al.’s study, a cohort of 18 OI patients (12 females, average age of 8.86 ± 3.90 years) was assembled. Among these patients, eight underwent a cyclic intravenous neridronate regimen. Each OI patient was meticulously paired with a healthy control of similar age and gender. The findings unveiled that both untreated and treated OI subjects exhibited significantly augmented serum levels of DKK1 and RANKL in contrast to controls. Notably, serum samples from patients with elevated DKK1 levels hindered osteoblast differentiation and the expression of OPG in vitro (Brunetti et al. 2016). In our prior investigation into the therapeutic potential of micro ribonucleic acid-29a (miR-29a) antagonists for treating OI in a murine animal model, we demonstrated the ability of pre-miR-29a to augment the local expression of DKK1 in OI-afflicted mice, which was subsequently restored to normal levels by anti-sense miR-29a (Ko et al. 2023a). These observations align intriguingly with the current study’s exploration into the therapeutic ramifications of DKK1-AS in OI mice. This congruence prompts the consideration of DKK1 inhibition as a promising avenue for therapeutic intervention in the realm of OI-related bone disease.

OI mice exhibited diminished bone mass and escalated DKK1 expression, paralleling the skeletal deterioration patterns evident in OI patients. Remarkably, treatment with DKK1-AS effectively ameliorated bone mass reduction and microstructural alterations in OI mice. It concomitantly enhanced trabecular histomorphometric characteristics, mineral accrual, ex vivo osteoblast differentiation potency, while attenuating bone resorption and ex vivo osteoclastogenesis in OI microenvironments. The reinstatement of skeletal homeostasis, orchestrated through the Wnt/β-catenin/TCF4 signaling axis, and orchestrated actions of RANKL and OPG, facilitated an augmentation of bone formation and resorption dynamics in OI skeletal tissues.

Collectively, this study underscores the involvement of Wnt/DKK1 interactions in dampened bone formation processes among OI patients. The analytical findings herein underscore the potential of abrogating Dkk-1’s deleterious influence as an adjunct molecular strategy for safeguarding against excessive bone mass compromise inherent to OI. These insights offer compelling impetus for the exploration of emerging Dkk-1 modulation strategies as a means to address severe skeletal deterioration within the OI patient cohort in the future.

The study has certain limitations. Firstly, T-score is a debatable intermediary criterion in OI, given the absence of more stringent severity criteria (e.g., Sillence type) for correlation with serum DKK1 levels in our study, as all participants belonged to type I OI. Secondly, we chose the B6 strain as the normal control mice over the controls proposed by Jackson Laboratory with the intention of selecting mice with entirely healthy bone structures. However, the selection of the B6 strain may introduce some heterogeneity in genetic background. Nevertheless, we do not suppose this will significantly confound the results, as the primary focus is on the effects of administering DKK1-AS or DKK1-S to OI mice in comparison to untreated OI mice. Thirdly, homozygous oim/oim mice employed in this study present a more severe, dominant type of OI, which does not entirely mirror the molecular pathology observed in human subjects with much milder type I OI (Saban et al. 1996). Fourthly, there is a deficiency in toxicity assessment and in vitro kinetic data, dose effect, alongside a scarcity of tolerance data in both wild-type and OI mice. Fifthly, while our study did not identify life-threatening renal or hepatic toxicity linked to DKK1-AS, the potential existence of unexpected off-target effects warrants consideration. Type I OI in humans is typically mild and characterized by a deficiency in collagen I with normal molecular profiles, and osteoblasts typically do not exhibit endoplasmic reticulum stress associated with high levels of misfolded proteins. Patients with type I OI typically have a normal lifespan, stature, and minimal skeletal deformities, with fractures typically occurring before puberty. These off-target effects of DKK1-AS are particularly concerning in individuals with milder forms of OI. Sixthly, DKK1-AS does not comprehensively address the various molecular pathologies linked to OI severity, including collagen insufficiency, dominant interference in assembly, and endoplasmic reticulum stress. Specifically, endoplasmic reticulum stress often manifests in severe OI types, where dominant interference in wild-type assembly is frequent. This stress leads to significant osteoblast apoptosis, contributing to the disease’s severe bone phenotype and lethality. Although DKK1-AS may enhance collagen I expression, it could potentially exacerbate the endoplasmic reticulum stress phenotype (Yuan et al. 2024). Therefore, further refinement of DKK1-AS to encompass all associated molecular pathologies in severe type OI is necessary. Finally, the genetic testing information is not comprehensive enough and is only available for three out of the nine recruited OI patients.

## Conclusions

Our research revealed an inverse link between serum DKK1 levels and lumbar spine and hip T-scores. Osteogenic genes in OI patient BMSCs exposed to Wnt3a recombinant protein exhibited higher expression than untreated or DKK1-treated BMSCs. Inferior micro-CT morphometric parameters associated with OI improved with DKK1-AS administration. DKK1-AS partially restored compromised osteogenic gene expression in OI mice’s BMSCs and mitigated reduced osteogenic capacity and heightened osteoclastogenesis in untreated OI mice. DKK1-AS reduced osteoclast counts in untreated OI mice’s bone tissue and reinstated decreased β-catenin and TCF4 expressions. DKK1-AS intervention improved MAR and BFR/BS values in OI bone tissues, also elevating OPG and suppressing RANKL expression. These outcomes highlight DKK1-AS’s potential in addressing OI-related bone challenges.

## Abbreviations

DKK1: Dickkopf-1
OI: Osteogenesis imperfecta
DKK1-AS: Dickkopf-1 antisense (DKK1-AS)
BMSC: Bone marrow stromal cell
DKK1-S: DKK1-sense
TCF4: T-cell factor 4 (TCF4)
MAR: Mineral apposition rate
BFR/BS: Bone formation rate per bone sur-face (BFR/BS)
OPG: Osteoprotegerin
RANKL: Nuclear factor-kappa B ligand
BMD: Bone mineral density
BV/TV(%): Trabecular bone volume to total volume fraction
Tb.Sp (mm): Trabecular separation
Tb.Th (mm): Trabecular thickness
Tb.N (1/mm): Trabecular number

## Declarations

### Availability of data and materials

Data sharing is not applicable to this article as no datasets were generated or analysed during the current study.
